# Quantifying autophagy using novel LC3B and p62 TR-FRET assays

**DOI:** 10.1371/journal.pone.0194423

**Published:** 2018-03-19

**Authors:** Alberto Bresciani, Maria Carolina Spiezia, Roberto Boggio, Cristina Cariulo, Anja Nordheim, Roberta Altobelli, Kirsten Kuhlbrodt, Celia Dominguez, Ignacio Munoz-Sanjuan, John Wityak, Valentina Fodale, Deanna M. Marchionini, Andreas Weiss

**Affiliations:** 1 IRBM Science Park, Pomezia, Rome, Italy; 2 IRBM Promidis, Pomezia, Rome, Italy; 3 Evotec AG, Manfred Eigen Campus, Hamburg, Germany; 4 CHDI Management/CHDI Foundation, New York, New York, United States of America; Northwestern University, UNITED STATES

## Abstract

Autophagy is a cellular mechanism that can generate energy for cells or clear misfolded or aggregated proteins, and upregulating this process has been proposed as a therapeutic approach for neurodegenerative diseases. Here we describe a novel set of LC3B-II and p62 time-resolved fluorescence resonance energy transfer (TR-FRET) assays that can detect changes in autophagy in the absence of exogenous labels. Lipidated LC3 is a marker of autophagosomes, while p62 is a substrate of autophagy. These assays can be employed in high-throughput screens to identify novel autophagy upregulators, and can measure autophagy changes in cultured cells or tissues after genetic or pharmacological interventions. We also demonstrate that different cells exhibit varying autophagic responses to pharmacological interventions. Overall, it is clear that a battery of readouts is required to make conclusions about changes in autophagy.

## Introduction

Macroautophagy is a cellular process that leads to the inclusion of cytoplasmic contents by double-membraned vesicles called autophagic vacuoles (AV; also called autophagosomes) and fusion to lysosomes for degradation, and is the prevalent process in a more general pathway; here we will refer to this process as autophagy throughout. Genetic studies in yeast have identified autophagy related genes that are essential to induction, formation or maturation of AVs, or degradation of substrates. LC3 is the best characterized mammalian core autophagy protein and plays an essential role in initiation and formation of AVs. LC3I is found in the cytoplasm and is conjugated to the lipid phosphatidylethanolamine to form LC3II, which binds to the AV membrane and can be used as a marker of AVs.[1] SQSTM1/p62 is a selective autophagy receptor, which sequesters ubiquitinated proteins into AVs by interacting with LC3.[2] In addition, p62 is a substrate for autophagic degradation, therefore its degradation can be used as a marker of autophagic clearance.[2–4]

Autophagy is normally induced under stress conditions, such as starvation, to produce energy from metabolites generated by the degradation of AV content,[5,6] after fusion with the lysosome.[7] Autophagy also clears the cell of damaged organelles, infectious agents, misfolded proteins, and protein aggregates.[8] Altered protein features, such as mutations that confer toxicity or increased propensity to aggregate, are hallmarks of neurodegenerative diseases, therefore, upregulating autophagy to clear AV content has been proposed as a viable therapeutic strategy for CNS disorders.[9] However, the role of autophagy in neurodegeneration is still controversial and requires further elucidation. Misfolded proteins observed in a number of neurodegenerative diseases have been associated with impaired autophagy, but it is unclear whether this is a direct consequence of the misfolded proteins or autophagic machinery.[7–12] There are several promising reports describing beneficial effects of autophagy upregulation in animal models of Alzheimer’s disease (AD),[13,14] Parkinson’s disease (PD),[15] spinocerebellar ataxia (SCA),[16] and Huntington’s disease (HD).[14–17]

Autophagy is a dynamic multi-faceted process that is technologically challenging to measure, therefore, investigating the role of misfolded proteins in autophagy or evaluating evidence for the therapeutic potential of modulating autophagy should use multiple readouts, at different timepoints when possible.[18] A limitation to further identifying causative links between autophagy and disease is that current autophagy assays are either based on reporters or unsuited to high-throughput screening (HTS); while the former assays are amenable to mid-throughput screening, introducing exogenous markers to cells may, in itself, alter autophagy.[19] Large genetic or pharmacological screens, based on endogenous readouts, have the potential to identify novel targets or molecules suitable for development of future therapeutic strategies.

Here we describe a panel of cell-based assays, amenable to HTS, that detect autophagy modulation through endogenous markers. We also present a genetic and pharmacological validation of the readouts and propose that these assays are suitable to miniaturization, as well as measuring autophagy modulation in primary cells and tissues.

## Materials and methods

### Cell cultures

HEK293T cells (from ATCC) were cultured in DMEM (Gibco, 31885023) supplemented with 10% FBS (Gibco, 26140079) and 1% penicillin and streptomycin (Gibco, 15140122) at 37°C, 5% CO_2_. For assays in 96-well plates, cells were seeded on plates coated with poly-L-lysine (Gibco, P4707) for 2 h at 37°C. Treatments with compounds started 24 h after plating.

Cultures of rat primary cortico-striatal neurons and astrocytes were prepared from E18 rat embryos. Briefly, cortex and striatum were dissected from the brain of embryos and dissociated to single cells by papain/DNAse treatment in neurobasal medium (Gibco, 21103049) supplemented with 4% B-27® (Gibco, 17504044), 0.5% penicillin and streptomycin, 2 mM GlutaMAX™ (Gibco, 35050–038) and 5 mM KCl. In order to obtain neuronal cultures, cortical and striatal cell suspensions were mixed at 1:1 ratio and seeded in tissue culture plates coated with 100 μg/ml poly-D-lysine (Gibco, P0899). After 3 DIV (days in vitro), 50% medium was changed. Treatment started at 4 DIV. To obtain astrocyte cultures, the dissected cortical cell preparation was seeded in T-75 flasks, in neurobasal medium supplemented with 4.4% qualified inactivated FBS, 2 mM GlutaMAX™, 10 mM KCl, 33 ml sterile water, 50 μg/ml gentamicin sulphate (Gibco, 15710–049) and 1% penicillin and streptomycin. Flasks were not poly-lysine coated in order to avoid neuronal adherence. Media was changed every 3 or 4 DIV after each passage, and primary astrocytes were purified by simply passaging them every 7 days until the third passage. Astrocytes were seeded to start experiments at the fourth passage.

### Compound treatments

Compound treatments were performed in 96-well clear plates (Greiner, 353072) and 96-well black plates to evaluate viability (Greiner, 655090). HEK293T cells (15,000 cells/well) were treated 24 h post seeding, while rat neurons and astrocytes were treated at 4 DIV post seeding (50,000 and 3,000 cells/well respectively). All tested compounds KU0063794 (Sigma-Aldrich, SML0382), NVP-TAE684, SU11652 and bafilomycin A1 (Sigma-Aldrich, B1793) were dissolved in DMSO and serially diluted for testing. The final amount of DMSO solutions tested in cell media never exceeded 0.25% v/v. 2, 6 or 24 hours post-treatment cells were PBS washed and lysed in 40 μl of lysis buffer per well: TBS, 0.4% Triton- X, protease inhibitors cocktail (Roche, 11836170001).

### Bafilomycin A1 co-treatment

Co-treatment experiments were carried out using rat astrocytes plated in 60 mm dishes (250,000 cells/dish). Cells were treated at 4 DIV with 10 μM KU0063794, 5 μM NVP-TAE684, 1 μM SU11652, and DMSO as control. 50 nM bafilomycin A1 was added after 2 hours of compound treatment for an additional 4 hours. Cells were collected using trypsin, centrifuged and lysed in lysis buffer. Lysates were used for western blot analysis and to perform p62 and LC3B TR-FRET assays.

### Tissue preparation

Tissue samples were weighed, transferred to Wheaton tissue-manual homogenizer and 1:10 w/v ice cold homogenization buffer (0.4% (v/v) TritonX-100, 138 mM NaCl, 2.7 mM KCl, 8 mM Na_2_HPO_4_, 1.5 mM KH_2_PO_4_, protease inhibitors cocktail complete ultra; Roche #05892970, phosphatase inhibitors cocktail PhosSTOP; Roche #04906837001) were added. Tissue was homogenized with 5 strokes. Crude homogenate was transferred into Eppendorf tubes and frozen for 1h or longer at -80°C. For experiments to test integrity of LC3BII and p62 after storage or repeated freeze-thaws, homogenates were made and tested before freezing; after storage at -80°C for 1h (0d), 1, 7, 14, 18 or 22d (1F/T); or after storage at -80°C for 1h, 1, 7, 14, 18 or 22d, followed by thaw on ice and freezing at -80°C for 1h (2F/T).

### Time-resolved fluorescence resonance energy transfer (TR-FRET) assays

TR-FRET assays were performed transferring a proper amount of cell lysate to a low volume 384 well plate (Greiner, 784080) and adding the antibody pairs diluted in lysis buffer. Terbium cryptate (Tb) LC3 (Sigma, L8918) and p62 (Abnova, H00008878-M01) antibodies and LC3 (Sigma, L7543) D2-fluorophore antibody were labeled by CisBio (Bagnols, France). The Alexa-647 labeled p62 antibody (Sigma, P0067) was generated using the Alexa Fluor®647 antibody labeling kit (Life Technologies, A20186) according to manufacturer's instructions.

For p62 detection, 5 μl of lysate was incubated with 0.03 ng/μl of p62-Tb antibody and 16.6 ng/μl of p62-Alexa-647 antibody. For LC3B detection, 10 μl of lysate was incubated with 0.18 ng/μl of LC3-Tb antibody and 23.3 ng/μl of LC3-D2 fluorophore antibody. TR-FRET measurements were routinely performed following incubation for 5 or 18 hours, for p62 and LC3 assays respectively, at room temperature, using an EnVision reader (Perkin Elmer) following excitation at 320 nm (time delay 100 msec, window 400 msec, 100 flashes/well). Values were collected as the ratio between fluorescence emission at 665 nm and 615 nm (665/615) and were expressed in percentage with respect to the DMSO treated controls.

Cell lysates, collected from 60 or 100 mm dishes, such as in bafilomycin A1 co-treatment experiments, were assayed in serial dilutions starting from 10 μg of total lysates for LC3B detection and 5 μg of total lysates for p62 detection. Obtained fluorescence ratios were fitted in curves using a four-parameter logistic (4PL) model using GraphPad Prism software. Fold increases in p62 and LC3IIB levels were evaluated by comparing EC_50_ values obtained from fitted curves with respect to the control (DMSO treated) samples. Recombinant p62 protein (Enzo life sciences) was used to measure sensitivity of p62 TR-FRET.

### Cell viability evaluation by immunofluorescence

Immunofluorescence in 96 well black plates (Greiner, 655090) was used to evaluate viability after cell treatment with each compound. Cells were PBS washed and fixed in 4% PFA, 4% sucrose at room temperature for 20 min. Fixed cells were first incubated with PBS, 1% donkey serum (Jackson Immunoresearch, 017-000-121), 1% BSA, 0.1% Triton X, for 1 hour at room temperature. HEK293T cell and astrocyte viability were evaluated by nuclei count after staining with 1μM H33342 in solution: PBS, 0.2% BSA, 0.05% Triton X. Neuron viability was evaluated by analyzing morphometric parameters, which are described by the ratio between the average of neurite length and the soma signal intensity (sum of the averages per well). Neurite length and soma signal intensity were determined after over-night labeling with anti-MAP2 (Biolegend, 822501; 1:5,000) in staining solution. The anti-chicken-Alexa Fluor®555 (Life Technologies, A-21437) was used as secondary antibody at 1:2,000 dilution, in staining solution for one hour. Immunostaining with primary anti-glial fibrillary acidic protein (Sigma, G9269; 1:1,000) was also performed as quality control for the neuronal preparation, using the secondary antibody anti-rabbit-Alexa Fluor®488 (Life Technologies, A-21206) at 1:2,000 dilution.

Images were acquired with IN Cell Analyzer 2000 from GE Healthcare, analyzing 9 fields per well with a 20X objective. Data analysis were carried out using IN Cell Developer Toolbox V1.9.3 from GE Healthcare.

### Western blot analysis and antibodies

Rat neurons and astrocytes were cultured 1 x 10^6^ and 250,000 cells/dish in 100 mm (Falcon, 353003) and 60 mm dishes (Falcon, 353004) respectively; HEK293T were seeded 600,000 cells/well in 6 well plates (Falcon, 353934). After treatment, cell cultures were lysed in lysis buffer (TBS, 0.4% Triton X, protease inhibitors cocktail); lysates were sonicated, clarified by centrifugation at 10,000 × g for 2 min at 4°C and evaluated for protein concentration using the BCA protein assay kit (Novagen) according to manufacturer's protocol. Depending on cell type, 10 to 30 μg of total lysates were denatured at 95°C in 4X loading buffer (125 mM TrisHCl pH 6.8, 6% SDS, 4 M urea, 4 mM EDTA, 30% glycerol, 4% β-mercaptoethanol and bromophenol blue) and loaded on NuPAGE® Novex® 4–12% bis-tris gel with precision plus protein standard marker (Biorad, 161–0364). Proteins were transferred on PVDF membranes using the Trans-Blot® Turbo™ transfer system (Biorad, 1704157) following manufacturer's protocol. Membranes were blocked 1 hour at room temperature in 1X TBS, 0.1% Tween, 5% non-fat milk and probed overnight at 4°C for LC3B (Cell Signaling, 2775, 1:1,000), p62 (Abnova, H00008878-M01, 1:400) and GAPDH (Sigma, G9545, 1:5,000). Membranes were washed and incubated with anti-mouse HRP (Sigma, A4416, 1:5,000) or anti-rabbit HRP (Sigma, A6154, 1:5,000) secondary antibodies. Protein bands were detected using the ECL substrate SuperSignal™ west femto maximum sensitivity (Life Technologies, 34095) at the Molecular Image Chemidoc XRS system from Biorad.

### Gene silencing, RNA purification and real-time quantitative PCR

HEK293T cells, seeded at 600,000 cells/well in 6 well plates, were transfected after 24 hours from seeding using SQSTM1 and MAP1LC3B shRNA plasmid DNA (from Sigma-Aldrich) and Lipofectamine® 2000 transfection reagent (Life Technologies 11668019) according to manufacturer's protocol. Cells were harvested 48 hours after transfection. RNA was purified using the MirVana PARIS RNA and native protein purification kit (Thermo Fisher AM1556), following the manufacturer’s instruction, which allowed purification of RNA and proteins from the same sample for real-time quantitative PCR, TR-FRET and western blot analyses. RNA was further purified from DNA traces by performing a treatment with TURBO DNA-free kit (Ambion, AM1907). Messenger RNA was retrotranscripted using the superscript III first-strand synthesis system for RT-PCR (Life Technologies 18080–051) according to manufacturer's protocol. The power SYBR green PCR master mix (ThermoFisher, 4367659) was applied to test p62 and LC3B expression with the T900 HT AbiPrism real-time PCR Instrument. Expression of p62 and LC3B were calculated and compared with the expression of β-actin through the 2^-ΔΔCT approach and expressed relative to the scramble group.

### Mouse studies

All mice were treated in accordance with the Guide for the Animal Care and Use of Laboratory Animals (National Research Council), and all procedures were approved by the Institutional Animal Care and Use Committee at PsychoGenics, Inc. 6 month C57BL/6J mice were dosed one time with vehicle or 10mg/kg p.o. mTOR inhibitor INK-128. The mTOR inhibitor was prepared in 0.5% carboxymethyl cellulose/0.05% tween 80 aqueous solution. 0.5 or 2 hours after oral gavage, mice were terminally anesthetized with pentobarbital and blood collected by cardiac puncture; liver was collected and flash-frozen in liquid nitrogen.

### Pharmacokinetic analysis

The concentrations of INK-128 in plasma and tissue were determined by extraction of the drug from matrix followed by LC-MS/MS analysis. Plasma was thawed and mixed with 4 volumes of acetonitrile containing 0.1% formic acid and internal standards, diclofenac and reserpine at 200 ng/mL. This mixture was then centrifuged and the supernatant diluted 1:1 with water into the analysis plate. The liver samples were thawed, mixed with 3 volumes of water and homogenized using a Precellys-24 (Bertin Technologies). Following homogenization, a known volume of homogenate was extracted in a manner similar to that of plasma. The extracts were analyzed by LC-MS/MS using calibration curves prepared by adding INK-128 to liver homogenates. The chromatographic separation was achieved using a C18 column (Kinetex XB-C18 100A) maintained at 40°C and mobile phase comprised of 2.5 mM ammonium formate (eluent A) and methanol (eluent B) with a gradient changing from 5% eluent B to 95% eluent B over 2 min. The peaks were monitored using a Waters Xevo_TQMS in the MRM mode with ESI + ionization at source temperature of 150°C and desolvation temperature of 500°C. The MS/MS transitions measured were 310.17→225.98 for INK-128, 296.05→214.14 for diclofenac, and 609.37 →195.09 for reserpine. The concentrations of INK-128 were determined based on peak areas normalized for the internal standard (diclofenac) peak area vs the peak areas of the appropriate calibration curve.

### Pharmacodynamic analysis

p62 and LC3II levels in liver samples of compound treated mice were quantified using crude liver tissue homogenates with a concentration of 0.6mg/ml total protein for p62 analysis and 3.5mg/ml for LC3II analysis. Phospho (Ser240/244) and total S6RP levels in tissue homogenates were analyzed according to manual using a commercial electrochemiluminescence kit (MSD, K15139D-1).

### Statistical analyses

Statistical significance was analyzed using Student’s t-test for experiments with only two groups. Analysis of variance (ANOVA) was performed for experiments with more than two groups. If statistical significance (p<0.05) was found, we performed post-hoc analysis for multiple comparisons. All graphs represent avg±SEM, unless ±SD is noted. GraphPad Prism software was used for all analyses.

## Results

### TR-FRET

**Fig 1:**
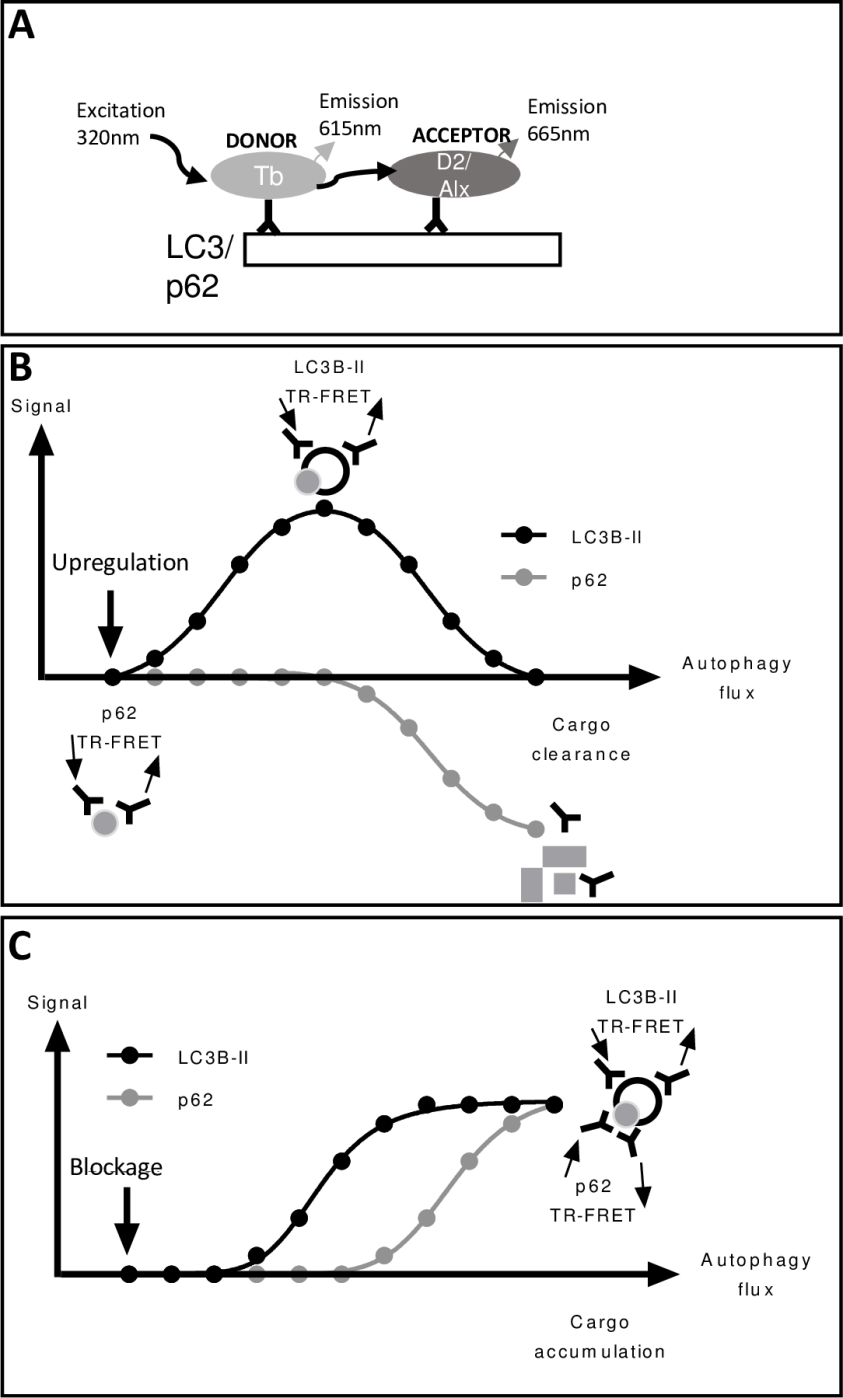
Overview of TR-FRET assay. The TR-FRET signal relies on transfer of energy between two fluorophores, a donor and acceptor, when in close proximity. When two labeled molecules come together, excitation of the donor by an energy source triggers energy transfer towards the acceptor, which emits a fluorescent signal. Tb labeled LC3II donor and D2 labeled LC3II acceptor antibodies or Tb labeled donor p62 and Alexa-647 labeled acceptor p62 antibodies were used (A). Compounds that upregulate autophagy induce close proximity of the LC3II donor and acceptor antibodies, resulting in an accumulation of signal as AVs are formed; that signal is reduced over time as AVs are turned over. P62 donor and acceptor antibodies come in close proximity as they find p62 proteins, the signal declines over time as p62 is degraded (B). Compounds that block autophagy at the lysosome have a different signal. The LC3II signal accumulates as the donor and acceptor antibodies come together in new AVs, but there is no degradation of the signal since the AVs are not degraded. Similarly, there is no turnover of the p62 signal (C).

TR-FRET is a homogeneous assay able to measure molecule proximity by fluorescence resonance energy transfer.[20] Tb labeled LC3II donor and D2 labeled LC3II acceptor antibodies or Tb labeled donor p62 and Alexa-647 labeled acceptor p62 antibodies were used (Fig 1A). Compounds that upregulate autophagy induce close proximity of the LC3II antibodies resulting in an accumulation of signal as AVs are formed; that signal is reduced over time as AVs are turned over. The p62 signal decreases over time as that substrate is degraded (Fig 1B). Compounds that block autophagy at the lysosome have a different signal. The LC3II signal accumulates as the donor and acceptor antibodies come together in new AVs, but there is no degradation of the signal since the AVs are not degraded. Similarly, there is no turnover of the p62 signal (Fig 1C).

### Technical development and evaluation of p62 and LC3B-II TR-FRET assay specificity

**Fig 2:**
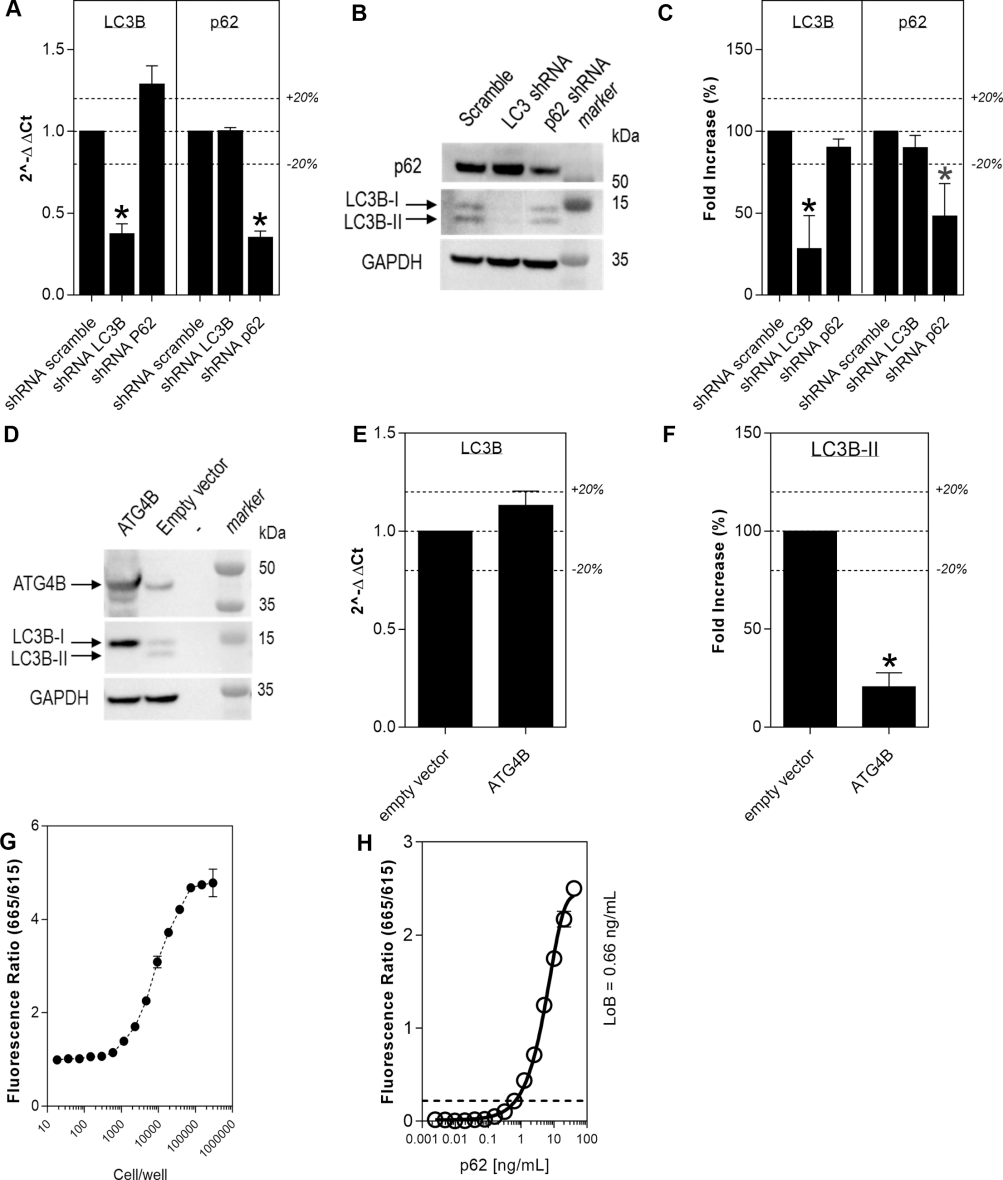
Evaluation of LC3B-II and p62 TR-FRET specificity. Genetic validation of the LC3B-II and p62 readouts was achieved by gene silencing of LC3B and p62 in HEK293T cells using shRNA. *LC3B* and *p62* mRNA were reduced after shRNA of each gene, compared to shRNA scramble, as verified by qRT-PCR (N = 3; avg±SD; Student’s t-test (unpaired; two-tailed); *p<0.001), expression levels were calculated using the 2^-ΔΔCT method and expressed relative to scramble control (A). A corresponding reduction of protein levels was observed by western blot (B) and TR-FRET (N = 4; avg±SD; Student’s t-test (unpaired; two-tailed); *p<0.005; expression relative to scramble control; C). ATG4B overexpression in HEK293T cells was confirmed with western blot (D). ATG4B overexpression did not alter LC3B mRNA levels as seen by qRT-PCR (N = 3, avg±SD; Student’s t-test (unpaired, two-tailed) p>0.05, expression levels were calculated using the 2^-ΔΔCT method and expressed relative to scramble control (E) but clearly reduced LC3B-II detection, as measured by TR-FRET (expressed relative to empty vector; N = 3, unpaired t-test, *p<0.001 (F) and western blot (D). LC3B-II quantification by TR-FRET (fluorescence ratio of 665/615 nm) in HEK293 cells showed detection with as few as 2000 cells/well (N = 2; avg±SD; G). Different concentrations of purified p62 were measured and the p62 TR-FRET assay is sensitive enough to detect 1ng/ml purified recombinant p62 protein (signal expressed as fluorescence ratio of 665/615 nm; N = 2; avg±SEM; H).

*LC3B* and *SQSTM1* (p62) gene silencing was performed on HEK293T cells in order to verify the specificity of the TR-FRET signal for the two proteins. Transfection of shRNAs specific for each target led to an approximate 60% decrease of mRNA for each transcript, with respect to scramble transfected samples (n = 3; *p<0.001; Student’s t-tests (unpaired; two-tailed)). Knockdown of LC3B did not influence p62 mRNA and knockdown of p62 did not influence LC3B mRNA (Fig 2A). There was a marked decrease in p62 and LC3B proteins as detected by western blot (Fig 2B). When protein levels of the two targets were quantified by TR-FRET, on the same lysates as above, there was a similar reduction in p62 and LC3B signal as observed by western blot, with respect to the scramble treated (n = 4; *p<0.005; Student’s t-tests (unpaired; two-tailed) Fig 2C).

To determine whether the TR-FRET assay specifically detects the lipidated form of LC3B (LC3B-II), HEK293T cells were transfected to overexpress ATG4B. ATG4B cleaves LC3B-II to generate non-lipidated LC3B (LC3B-I); therefore, overexpression of this enzyme should abolish LC3B-II from cells. ATG4B overexpression in HEK293T cells was verified by western blot (Fig 2D). No modulation of *LC3B* gene expression was observed by qRT-PCR (n = 3; Student’s t-test (unpaired; two-tailed), p>0.05, Fig 2E), while a clear decrease of LC3B-II levels was detected by western blot together with an increase in LC3B-I intensity (Fig 2D). Likewise, there was a significant 80% reduction in LC3B TR-FRET signal detected on the same ATG4B overexpressing cell lysate (n = 3, Student’s t-test (unpaired; two-tailed), *p<0.001; Fig 2F), thereby demonstrating specificity of the assay for LC3B-II. To further validate the specificity and sensitivity of the LC3B TR-FRET assay, LC3B levels were measured in HEK293T cells plated at different densities over time (n = 2; avg±SD; Fig 2G). To further validate the p62 TR-FRET assay, different concentrations of purified p62 were used to assess the sensitivity of the p62 TR-FRET assay (n = 2; avg±SEM; Fig 2H).

Varying concentrations of salt, TritonX, Tween20 and glycerol were surveyed to identify the optimal lysis buffer to yield the most robust endogenous LC3 signal in HEK cells (n = 2; avg±SD; S1 Fig).

### Reference compound profiling on HEK293T and primary cultures of rat neurons and astrocytes

**Fig 3:**
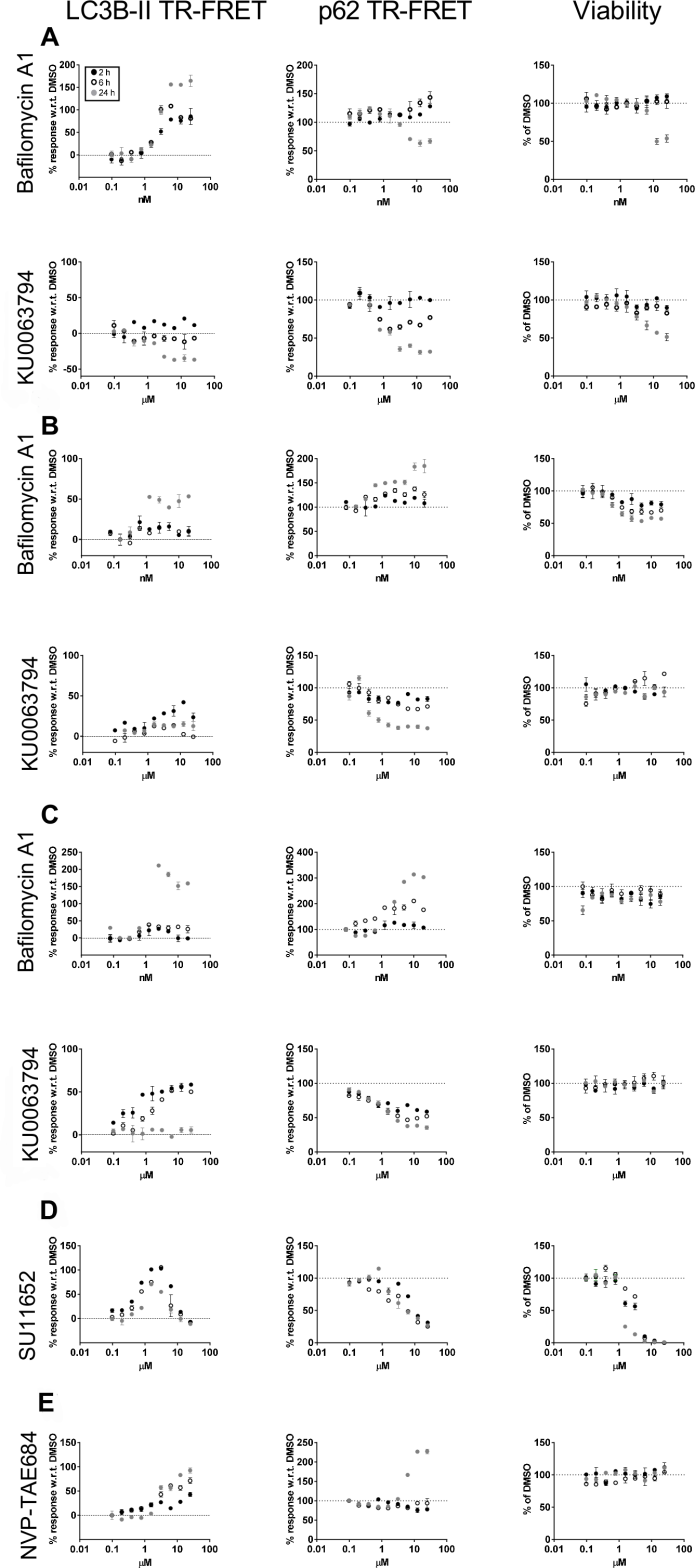
LC3B-II and p62 quantification in response to tool compounds treatment. HEK293T cells (A), rat cortico-striatal neurons (B) and rat astrocytes (C) were treated with a serially diluted autophagy inhibitor (bafilomycin A1) or upregulator (KU0063794) and examined at 2, 6 and 24 hours post-treatment. LC3B-II and p62 were measured with TR-FRET (A-C). The response to compound treatment is reported as percentage average of three replicates with respect to vehicle (DMSO) treated samples (100%). Cell viability was evaluated by H33342 stained nuclei count for HEK293T (A) and astrocytes (C-E) and by neurite length/soma (morphometric readout) on MAP2 stained neurons (B). Astrocytes were also treated with SU11652 and NVP-TAE684 and LC3B-II, p62 and viability were measured with TR-FRET (D-E). Each data point is the mean±SEM (N = 3).

**Fig 4:**
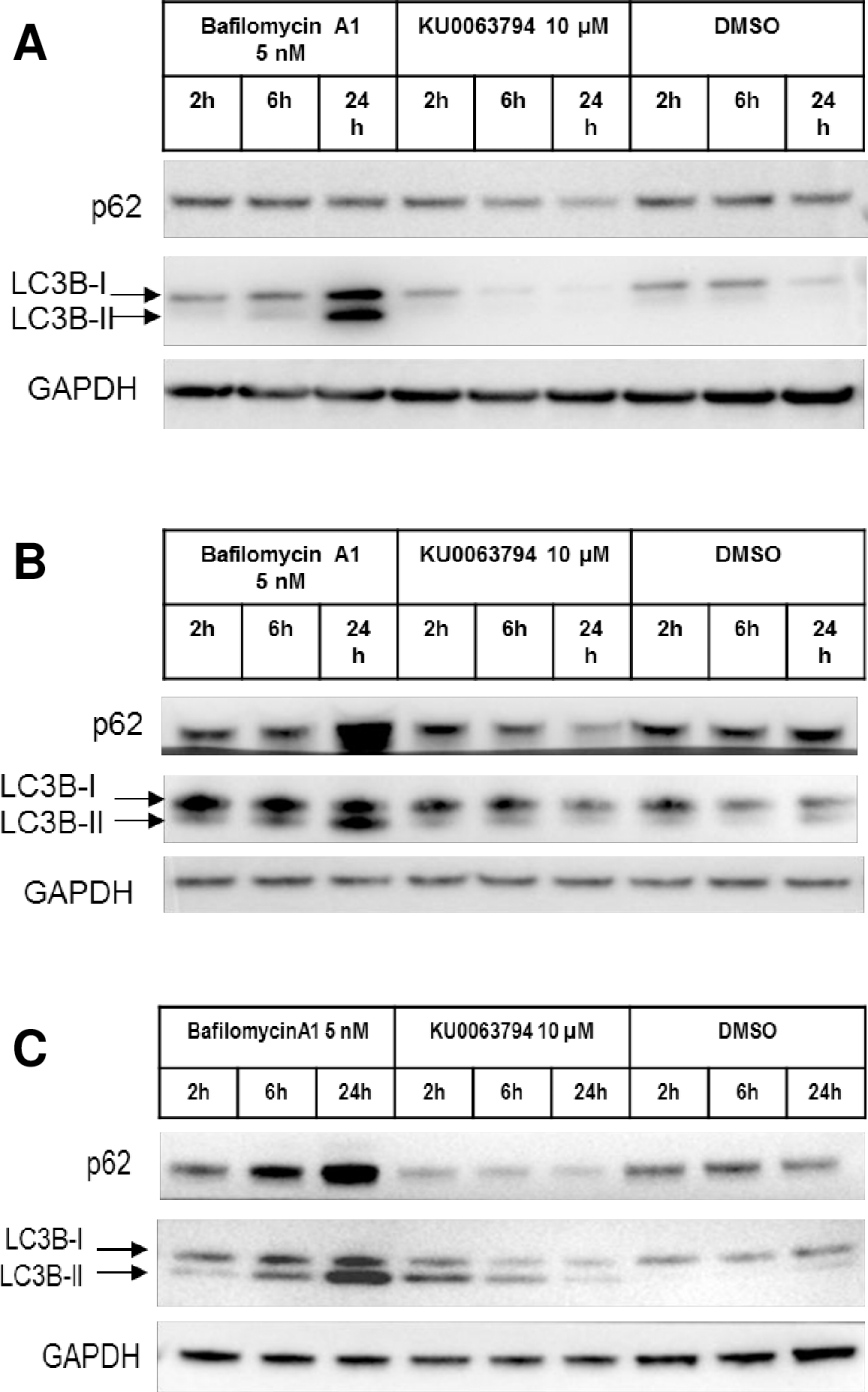
LC3B-II and p62 protein responses after tool compound. HEK293T cells (A), rat cortico-striatal neurons (B) and rat astrocytes (C) were treated with an autophagy inhibitor (5 nM bafilomycin A1) or upregulator (KU0063794) and examined at 2, 6 and 24 hours post-treatment, compared to DMSO. Western blot analysis is presented for LC3B-I/II and p62 levels, with a GAPDH loading control.

To demonstrate the utility of p62 and LC3B-II quantification by TR-FRET, compounds previously reported to modulate the autophagy pathway were evaluated in different cell models. Bafilomycin A1[21] is an autophagy blocker, it inhibits vacuolar H+ ATPase (V-ATPase) and hinders the fusion between AVs and lysosomes; as a consequence, AVs accumulate since autophagic flux is inhibited. KU0063794[22] is an autophagy inducer which acts earlier in the pathway by inhibiting the mammalian target of rapamycin complex 1 and 2 (mTORC1/mTORC2). These two compounds were tested in serial dilutions starting at 25 nM for bafilomycin A1 and 25 μM for KU0063794. The study was carried out at 2, 6 and 24 hours post-treatment to potentially capture multiple cellular states in response to autophagy modulation. In HEK293T cells, bafilomycin A1 treatment resulted in a dose and time-dependent increase of LC3B-II levels with respect to vehicle (DMSO) treated cells (Fig 3A). A similar behavior was detected for p62 at 2 and 6 hours post-treatment. Bafilomicyin A1 concentrations higher than 2 nM resulted in cytotoxicity at 24 hours. The profile of LC3B-II and p62 at higher concentrations at 24 hours could be influenced either by non-autophagy related events and/or a decreased amount of cells in the testing sample, thereby making interpretation difficult. Using this assay, autophagy cannot be measured when viability is compromised. In HEK293T cells the LC3B-II TR-FRET signal increased in a dose-dependent manner at 2 hours post treatment with KU0063794 and then decreased with time, relative to the DMSO treated cells (Fig 3A). This behavior may be due to exhaustion of the cells ability to synthesize new AVs after an initial activation. Conversely, p62 decreased with time and KU0063794 concentration. As previously noted for bafilomycin A1 treatment, cell viability is influenced by the compound at 24 hours post treatment, therefore LC3B-II and p62 results at this time point must be taken with caution, especially since the p62 reduction appears to reduce in parallel with cytotoxicity. Western blots with LC3B and p62 confirmed what was observed with TR-FRET (Fig 4A). Bafilomycin A1 and KU0063794 were tested in primary rat cortical-striatal neurons and astrocytes. In contrast to HEK293T cells, these cells are post-mitotic (neurons) or slowly proliferating (astrocyte doubling time *in vitro*, with the present culture conditions, was estimated to be 7 days). Neurons and astrocytes were treated with serial dilutions of compounds starting at 25 nM for bafilomycin A1 and 25 μM for KU0063794. Nuclei count was used to assess the viability of astrocytes upon compound treatment, but was not suitable for neuronal cultures due to difficulty in segmentation by image analysis of clusters of neuronal nuclei. To overcome this limitation, viability of cultured neurons was evaluated by MAP2 labeled neurite length per soma. Bafilomycin A1 treatment in neurons caused an increase in both p62 and LC3B-II levels in a dose and time dependent manner, although the neuronal LC3B-II response to bafilomycin A1 was not very robust at concentrations that were not cytotoxic (Fig 3B). Similar to what was observed in HEK293T cells, a clear cytotoxic effect increased in magnitude over time. KU0063794 induced a short-lived and modest increase in LC3B-II at 2 hours and a time and dose-dependent reduction in p62, as measured by TR-FRET and western blot, in the absence of overt cytotoxicity (Figs 3B and 4B). When tested on astrocytes, bafilomycin A1 increased LC3B-II and p62, in a dose and time-dependent manner as determined by TR-FRET and western blot. Astrocytes had a robust response to KU0063794, increasing LC3B-II at 2 hours and decreasing p62 by 24 hours in the absence of cytotoxicity, suggesting upregulation of autophagy (Figs 3C and 4C). While the p62 and LC3B-II TR-FRET assays can detect changes in autophagy, it is clear that autophagy modulation differs depending on cell type. To highlight the importance of considering all 3 readouts when determining the state of autophagy, astrocytes were tested with 2 additional compounds, SU11652 and NVP-TAE684. Although SU11652 upregulated the LC3B-II TR-FRET signal and reduced the p62 TR-FRET signal, there is clear cytotoxicity at concentrations higher than 1μM, therefore this compound may upregulate autophagy at low concentrations and at higher concentrations cannot be interpreted. It is notable that the p62 signal appears to track with cytotoxicity (Fig 3D). NVP-TAE684 induced an increase in the LC3B-II signal in the absence of toxicity. However, there is no reduction in p62 and even an increase in p62 at higher concentrations at 24 hours, this suggests that the accumulation of AVs may be due to blockage of autophagy with this compound (Fig 3E).

### Confirmation of autophagy inducers vs blockers

**Fig 5:**
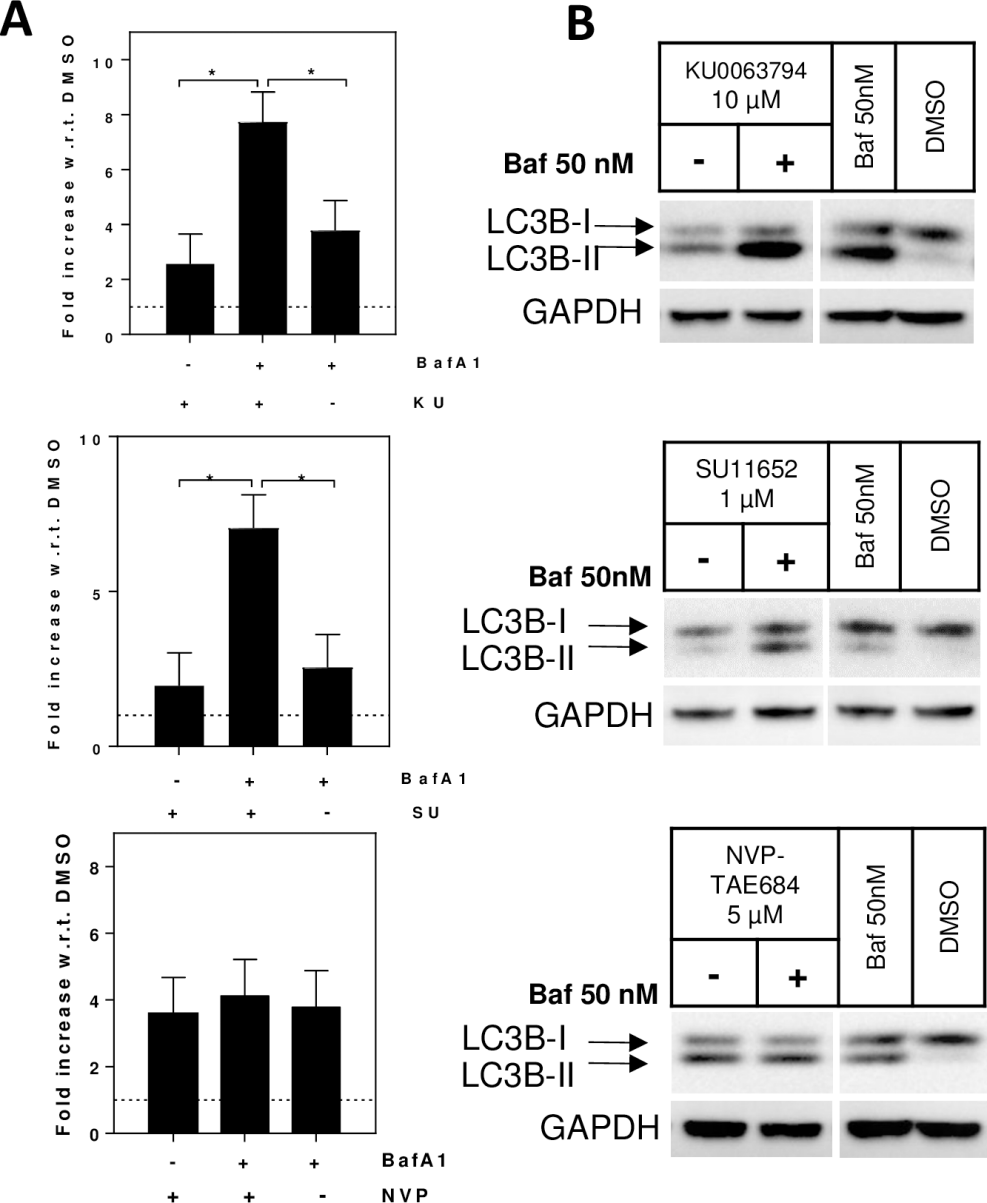
Co-treatment with bafilomycin A1 to distinguish autophagy enhancers versus blockers. Rat primary astrocytes were treated with 10 μM KU0063794, 1 μM SU11652 or 5 μM NVP-TAE684 (concentration selected from information in Fig 2) followed by either 50 nM bafilomycin A1 (+Baf) or vehicle (DMSO, -Baf) for an additional 4 hours. Control samples were treated with vehicle (DMSO) for 2 hours followed by either bafilomycin A1 (50 nM) or vehicle (DMSO) for an additional 4 hours. LC3B-II TR-FRET signals are reported as fold increase with respect to the vehicle. Co-treatment of KU0063794 and bafilomycin A1 increased LC3B-II, compared to KU0063794 or bafilomycin A1 alone (N = 2, one-way ANOVA, p<0.01; Tukey’s multiple comparison test, *p<0.05); co-treatment of SU11652 and bafilomycin A1 increased LC3B-II, compared to SU11652 or bafilomycin A1 alone (N = 2, one-way ANOVA, p<0.01; Tukey’s multiple comparison test, *p<0.05); co-treatment of NVP-TAE684 and bafilomycin A1 did not alter the LC3B-II signal (N = 2, one-way ANOVA, p>0.05; (A). Western blots (B) confirm the TR-FRET data.

The accumulation of AVs can be a result of either upregulated or blocked autophagy. To discriminate autophagy inducers from blockers LC3B-II and p62 quantification were measured in astrocytes co-treated with tool compounds and bafilomycin A1. In the presence of an autophagy blocker the accumulation of LC3B-II is not influenced by the addition of bafilomycin A1, as the effect of the two compounds are identical. Conversely, a compound able to upregulate autophagy induces the production of LC3B-II; as a consequence the blockage of such induced autophagy flux by bafilomycin A1 results in higher LC3B-II levels than compound alone. To perform this study we first identified a non-cytotoxic concentration of each tool compound that leads to an accumulation of AVs (Fig 3). Astrocytes were treated for two hours by one non-cytotoxic concentration of each of three compounds (10 μM KU0063794; 1 μM SU11652 and 5 μM NVP-TAE684) followed by four hours of either 50 nM bafilomycin A1 or vehicle (DMSO). DMSO only and 50 nM bafilomycin A1 only treated cells were also produced as control samples. Cell lysates of all the treatment conditions were analyzed both by TR-FRET and western blot. Co-treatment of KU0063794 and bafilomycin A1 resulted in an augmented LC3II-B signal, compared to KU0063794 or bafilomycin A1 treatment alone (N = 2, one-way ANOVA, Tukey’s multiple comparison test, *p<0.05), which is in line with literature reporting KU0063794 as an autophagy inducer[23] (Fig 5). The second compound is a pan-kinase inhibitor (SU11652)[18], co-treatment with bafilomycin A1 augmented the increase in LC3B-II, compared to SU11652 or bafilomycin A1 alone, suggesting that this compound is an autophagy inducer at low concentrations (N = 2, one-way ANOVA, Tukey’s multiple comparison test, *p<0.05; Fig 5). Lastly, co-treatment of the anaplastic lymphoma kinase (ALK) inhibitor NVP-TAE684[24] with bafilomycin A1 did not alter the LC3B-II signal, compared to NVP-TAE684 or bafilomycin A1 alone, confirming that this compound is an autophagy blocker (N = 2, one-way ANOVA, p>0.05; Fig 5).

### Quantification of autophagy in tissues

**Fig 6:**
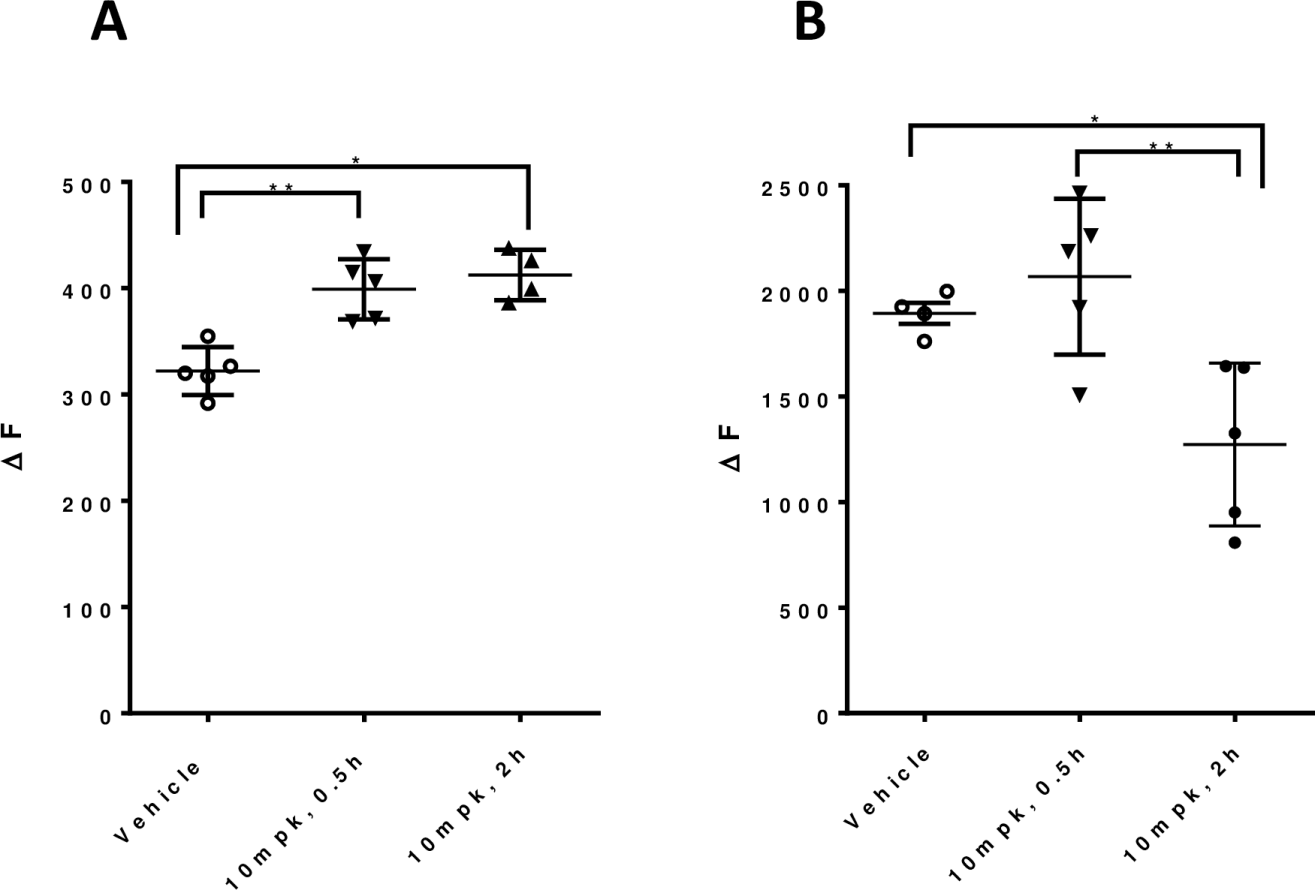
*In vivo* mTOR inhibition resulted in a measurable stimulation of autophagy. 6 month mice were treated one time with a mTOR inhibitor and sacrificed 0.5 or 2 hours afterwards. mTOR inhibition increased LC3B-II (A) and reduced p62 (B) levels in the mouse liver, as measured by TR-FRET and expressed as the fluorescence ration (665/615 nm) ΔF; N = 5; one-way ANOVA, p<0.01; Tukey’s multiple comparison test, *p<0.05, **p≤0.01.

The LC3B-II and p62 TR-FRET assays allow for label-free quantification of autophagy changes in tissues. In comparison to western blot, the TR-FRET assays are more quantitative and have higher throughput capabilities. As a proof-of-concept of the utility of the LC3B-II and p62 TR-FRET assays we used an mTOR inhibitor to demonstrate autophagy changes can be measured *in vivo* in a mouse. Mice were dosed with vehicle or 10 mg/kg mTOR inhibitor. Pharmacokinetic analysis revealed that the compound reached the tissue of interest (N = 5; one-way ANOVA, p<0.0001, Part A in S2 Fig). To confirm that mTOR was inhibited, we demonstrated that S6, a phosphorylation target of mTOR, was significantly inhibited (N = 5; one-way ANOVA, *p<0.01, analysis with outlier identification by Grubbs (alpha = 0.2); *p<0.01, Part B in S2 Fig). We further show that mTOR inhibition results in an increase in LC3B-II (N = 5; one-way ANOVA, *p<0.01; Tukey’s multiple comparison test *p<0.01, **p<0.05) and a reduction in p62 levels (N = 5; one-way ANOVA, *p<0.01; Tukey’s multiple comparison test *p<0.01; Fig 6), thereby demonstrating the utility of these assays in tissues.

We also examined the impact of storage or freeze thaws on brain homogenates; LC3B-II, but not p62 levels, were influenced by storage time (N = 4; one-way ANOVA, p<0.001; Tukey’s multiple comparison test *p<0.01, Part A in S3 Fig). The LC3B-II signal was only decreased at day 1, but no other time point, suggesting the signal may be more readily influenced by homogenization technique than storage. Storage or freeze thaws do not alter LC3B-II levels in HEK cells (N = 4; one-way ANOVA, p>0.05, Part B in S3 Fig) indicating a highly reproducible and robust protein quantification assay protocol for the autophagy marker proteins in cell lysates.

## Conclusions

We set out in these studies to establish a set of assays that could measure autophagy by quantifying endogenous markers, and could be used variously to screen in 384 wells as well as in primary cells and animal studies. Current methods to measure autophagy are extensively reviewed elsewhere[12]; TR-FRET offers the advantage of not relying on overexpression of a LC3B or p62 reporter, and is amenable for screening. Other reports use TR-FRET to measure autophagy, however, they rely on different antibody pairs (for example LC3-ATG4 or LC3-LAMP)[25,26] or require cells to express a GFP tagged autophagy reporter.[27] The approach outlined here, measures lipidated LC3B without the need for a reporter tag. We developed two TR-FRET assays based on antibody-mediated recognition of two primary markers, LC3B-II and p62; these two readouts, together with cell viability for cell-based assays, generated data relevant to interpreting autophagy modulation. In the cells tested in these studies, pharmacologically-induced autophagy upregulation is characterized by a rapid increase from a low basal level of LC3B-II, followed by a return to initial levels. As noted in other studies, the accumulation of LC3B-II reflects binding to AVs, which may reflect either upregulated autophagy or blockage of AV degradation,[18] making interpretation of LC3C-II levels alone difficult. In our studies, p62 decreases at later time points probably because it is a substrate for autophagic degradation.[3,28] Reduction of p62 may reflect upregulated autophagic clearance because it is a substrate for autophagy; but since p62 is involved in a number of different pathways [29–31] and is a promiscuous marker, its levels should not be measured in isolation. Our studies suggest that the combination of reduced p62, increased LC3B-II and absence of cytotoxicity -taken together at multiple time points and concentrations- can serve to identify upregulation of autophagy.

We demonstrate here that the signal produced by the two TR-FRET assays was selective in a cell lysate matrix by silencing the two targets: LC3B and p62. With regard to LC3B-II, the specificity of its detection with respect to the non-lipidated form was evaluated by overexpression of ATG4B, an enzyme that catalyzes the removal of the lipid moiety from LC3B-II[32]; we saw a decreased LC3B-II TR-FRET signal in ATG4B-overexpressing cells in the absence of any transcriptional modulation of LC3B, validating the specificity for the post-translational modification. We further validated the suite of assays by treatment with KU0063794 (mTOR inhibitor) and bafilomycin A1 (AVs-lysosome fusion inhibitor), which are known to induce and block autophagy, respectively.[21,23] We conducted this study on a proliferating cell line (HEK293T) and on primary cortico-striatal neurons and astrocytes derived from rat embryos. LC3B-II and p62 levels in each cell type were generally in agreement with the above described kinetic change of LC3B-II and p62. However, under the culturing conditions used, astrocytes produced the most robust response to autophagy stimuli. Furthermore, in the same concentration range a clear influence of cell viability of the two markers is detectable in HEK293T cells, especially at 24 hours post treatment. Assay results for unknown compounds should be interpreted carefully when cytotoxicity is evident.

We also compared measurement of LC3B-II using TR-FRET versus western blotting. One common method to discriminate between autophagy inducers and blockers is to block autophagy by bafilomycin A1 a few hours after treatment with the compound under investigation and evaluate the amount of LC3B-II by western blot. As demonstrated with KU0063794, an autophagy inducer results in an increase in LC3B-II, while co-treatment with bafilomycin A1 augments the signal, since the additional AVs that were made are not degraded by the lysosome. In contrast, NVP-TAE684, an autophagy blocker results in an increase in LC3B-II, but the signal is not altered by bafilomycin A1; additional blockage had no influence on the LC3B-II signal since there was no upregulation of AV generation. In both cases the TR-FRET and western blot gave similar results. The TR-FRET method is highly quantitative with its direct measurement of fluorescent emission, in comparison to western blots which are more qualitative and require a secondary antibody for visualization. It is noteworthy that the combination of the LC3B-II and p62 TR-FRET signals, plus cytotoxicity, predicts whether a compound is an autophagy stimulator or inhibitor, similar to bafilomycin A1 co-treatment.

In conclusion, we demonstrate here that evaluation of LC3B-II and p62 by TR-FRET (plus cell viability data) carried out in multiple doses and time points can provide insight into a compound’s ability to modulate autophagy, together with the concentration range at which the modulation is not accompanied by cytotoxicity. Notably, our assays have no genetic modification/reporting system, making them suitable not only to investigate non-transformed cell types but also applicable as a pharmacodynamic readout for *in vivo* studies.

## Supporting information

S1 Fig LC3B-II TR-FRET lysis buffer optimization. HEK293 cells were lysed in PBS buffers with different detergent (Tween20, Triton-X), ionic strength (NaCl) and glycerol concentrations. The strongest LC3B-II signals, expressed as fluorescence ratio (665/615 nm), were obtained with mild lysis buffer- Triton-X without NaCl or glycerol (N = 2, avg±SD).
(PDF)

S2 Fig Pharmodynamics of mTOR inhibitor. Oral gavage of 10mg/kg (mpk) mTOR inhibitor resulted in measurable detection of the compound in the plasma and liver, compared to vehicle treated (N = 5; one-way ANOVA, *p<0.0001, A). Target engagement was measured by a reduction in phosphorylation of S6, as presented by the ratio of phosphorylation over total S6 (N = 5; one-way ANOVA, p<0.01, analysis with outlier identification by Grubbs (alpha = 0.2); *p<0.01, B).
(PDF)

S3 Fig Evaluation of the storage of LC3B-II and p62 protein in cell lysates and mouse brain homogenates. Bulk amounts of brain homogenates (A) or HEK293T cell lysates treated with or without bafilomycin A1 (B) were distributed into aliquots. Aliquots of freshly generated homogenates and lysates were subjected to 1 freeze/thaw cycle (F/T) or 2 F/T cycles and were analyzed for BCA total protein, LC3II and p62 TR-FRET levels. No influence of up to two freeze-thaw-cycles and up to 3 weeks storage at -80°C on total protein, LC3B-II or p62 levels was observed in HEK cells (N = 4; one-way ANOVA, p>0.05), indicating a highly reproducible and robust protein quantification assay protocol for the autophagy marker proteins in cell lysates. Data is presented as the difference between bafilomycin A1 treated and untreated cells. LC3B-II, but not p62 levels, were influenced by storage time (N = 4; one-way ANOVA, p<0.001; Tukey’s multiple comparison test *p<0.01, in comparison to all other times).
(PDF)
