# Supplementary material for: Quantifying autophagy using novel LC3B and p62 TR-FRET assays
Source: PLoS One. 2018 Mar 19;13(3):e0194423. doi: 10.1371/journal.pone.0194423 (PMC5858923; doi:10.1371/journal.pone.0194423)
Supplement: S1 Fig — HEK293 cells were lysed in PBS buffers with different detergent (Tween20, Triton-X), ionic strength (NaCl) and glycerol concentrations. The strongest LC3B-II signals, expressed as fluorescence ratio (665/615 nm), were obtained with mild lysis buffer- Triton-X without NaCl or glycerol (N = 2, avg±SD). (PDF) [file pone.0194423.s001.pdf]

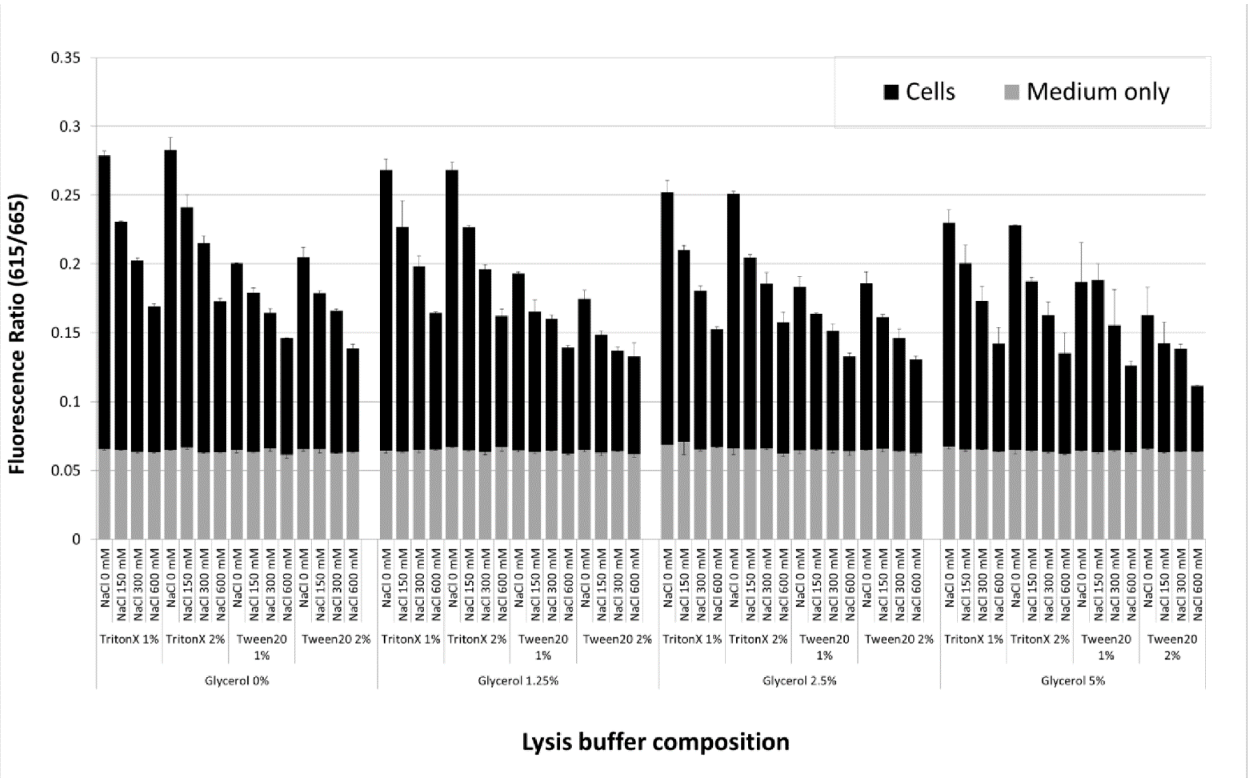

S1 Fig. LC3B-II TR-FRET lysis buffer optimization. HEK293 cells were lysed in PBS buffers with different detergent (Tween20, Triton-X), ionic strength (NaCl) and glycerol concentrations. The strongest LC3B-II signals, expressed as fluorescence ratio (665/615 nm), were obtained with mild lysis buffer- Triton-X without NaCl or glycerol (N=2, avg±SD).
