# Supplementary material for: Quantifying autophagy using novel LC3B and p62 TR-FRET assays
Source: PLoS One. 2018 Mar 19;13(3):e0194423. doi: 10.1371/journal.pone.0194423 (PMC5858923; doi:10.1371/journal.pone.0194423)
Supplement: S2 Fig — Oral gavage of 10mg/kg (mpk) mTOR inhibitor resulted in measurable detection of the compound in the plasma and liver, compared to vehicle treated (N = 5; one-way ANOVA, *p<0.0001, A). Target engagement was measured by a reduction in phosphorylation of S6, as presented by the ratio of phosphorylation over total S6 (N = 5; one-way ANOVA, p<0.01, analysis with outlier identification by Grubbs (alpha = 0.2); *p<0.01, B). (PDF) [file pone.0194423.s002.pdf]

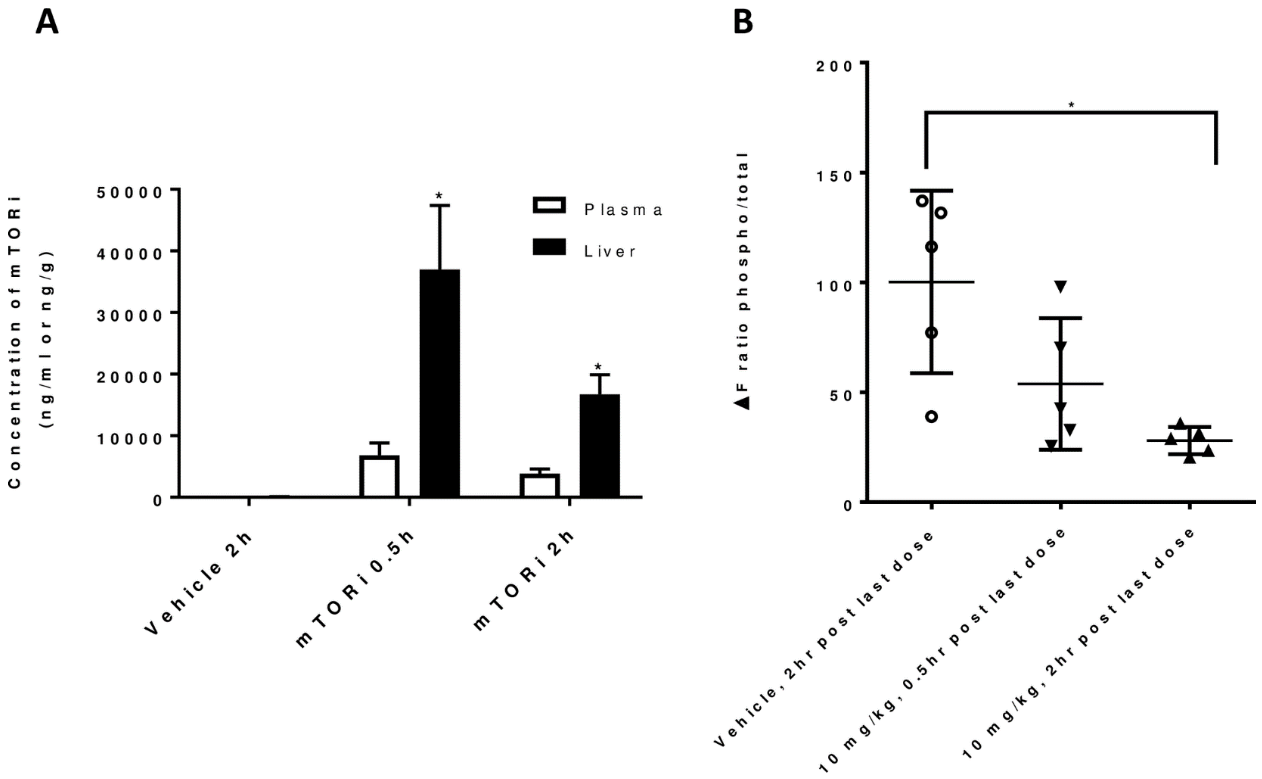

S2 Fig. Pharmodynamics of mTOR inhibitor. Oral gavage of 10mg/kg (mpk) mTOR inhibitor resulted in measurable detection of the compound in the plasma and liver, compared to vehicle treated (N=5; one-way ANOVA, *p<0.0001, A). Target engagement was measured by a reduction in phosphorylation of S6, as presented by the ratio of phosphorylation over total S6 (N=5; one-way ANOVA, p<0.01, analysis with outlier identification by Grubbs (alpha=0.2); *p<0.01, B).
