# Supplementary material for: Quantifying autophagy using novel LC3B and p62 TR-FRET assays
Source: PLoS One. 2018 Mar 19;13(3):e0194423. doi: 10.1371/journal.pone.0194423 (PMC5858923; doi:10.1371/journal.pone.0194423)
Supplement: S3 Fig — Bulk amounts of brain homogenates (A) or HEK293T cell lysates treated with or without bafilomycin A1 (B) were distributed into aliquots. Aliquots of freshly generated homogenates and lysates were subjected to 1 freeze/thaw cycle (F/T) or 2 F/T cycles and were analyzed for BCA total protein, LC3II and p62 TR-FRET levels. No influence of up to two freeze-thaw-cycles and up to 3 weeks storage at -80°C on total protein, LC3B-II or p62 levels was observed in HEK cells (N = 4; one-way ANOVA, p>0.05), indicating a highly reproducible and robust protein quantification assay protocol for the autophagy marker proteins in cell lysates. Data is presented as the difference between bafilomycin A1 treated and untreated cells. LC3B-II, but not p62 levels, were influenced by storage time (N = 4; one-way ANOVA, p<0.001; Tukey’s multiple comparison test *p<0.01, in comparison to all other times). (PDF) [file pone.0194423.s003.pdf]

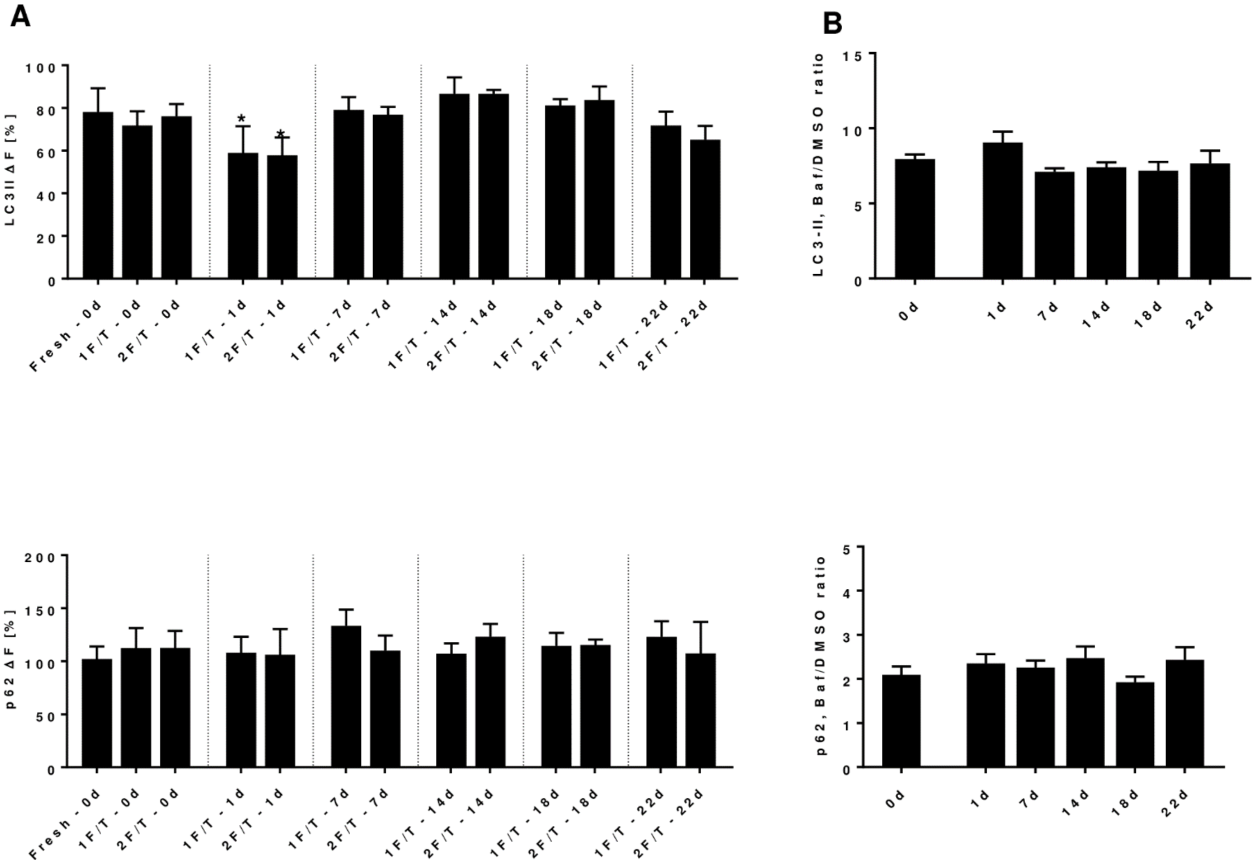

S3 Fig. Evaluation of the storage of LC3B-II and p62 protein in cell lysates and mouse brain homogenates. Bulk amounts of brain homogenates (A) or HEK293T cell lysates treated with or without bafilomycin A1 (B) were distributed into aliquots. Aliquots of freshly generated homogenates and lysates were subjected to 1 freeze/thaw cycle (F/T) or 2 F/T cycles and were analyzed for BCA total protein, LC3II and p62 TR-FRET levels. No influence of up to two freeze-thaw-cycles and up to 3 weeks storage at -80°C on total protein, LC3B-II or p62 levels was observed in HEK cells (N=4; one-way ANOVA, $p>0.05$), indicating a highly reproducible and robust protein quantification assay protocol for the autophagy marker proteins in cell lysates. Data is presented as the difference between bafilomycin A1 treated and untreated cells. LC3B-II, but not p62 levels, were influenced by storage time (N=4; one-way ANOVA, $p<0.001$; Tukey's multiple comparison test *$p<0.01$, in comparison to all other times).
